# Predicting acute ischemic stroke using the revised Framingham stroke risk profile and multimodal magnetic resonance imaging

**DOI:** 10.3389/fneur.2023.1264791

**Published:** 2023-09-28

**Authors:** Jiali Sun, Ying Sui, Yue Chen, Jianxiu Lian, Wei Wang

**Affiliations:** ^1^Department of MRI Room, First Affiliated Hospital of Harbin Medical University, Harbin, China; ^2^Department of Advisory Clinical Scientist C&TS North, Philips Healthcare, Beijing, China

**Keywords:** atherosclerosis, plaque, cerebral perfusion, high-resolution vascular wall imaging, revised Framingham stroke risk profile (FSRP) atherosclerosis

## Abstract

**Background and purpose:**

Patients with transient ischemic attacks (TIA) have a significant risk of developing acute ischemic strokes (AIS), emphasizing the critical need for hierarchical management. This study aims to develop a clinical-imaging model utilizing multimodal magnetic resonance imaging (mMRI) and the revised Framingham Stroke Risk Profile (FSRP) to predict AIS and achieve early secondary prevention.

**Methods:**

mMRI scans were conducted on patients with symptomatic intracranial atherosclerotic disease (ICAD) to assess vascular wall features and cerebral perfusion parameters. Based on diffusion-weighted imaging (DWI), patients were divided into two groups: TIA and AIS. Clinical data were evaluated to calculate the FSRP score. Differences in clinical and imaging characteristics between the groups were analyzed, and a predictive model for AIS probability in patients with ICAD was established.

**Results:**

A total of 112 TIA and AIS patients were included in the study. The results showed that the AIS group had higher proportions of FSRP-high risk, hyperhomocysteinemia, and higher value of low-density lipoprotein (LDL), standardized plaque index (SQI), and enhancement rate (ER) compared to the TIA group (*p* < 0.05). Mean transit time (MTT) and time to peak (TTP) in the lesion area were significantly longer in the AIS group (*p* < 0.05). Multivariate analysis identified FSRP-high risk (*p* = 0.027) and high ER (*p* = 0.046) as independent risk factors for AIS. The combined clinical and mMRI model produced an area under the curve (AUC) of 0.791 in receiver operating characteristic (ROC) analysis. The constructed nomogram model combining clinical and mMRI features demonstrated favorable clinical net benefits.

**Conclusion:**

FSRP-high risk and high ER were confirmed as independent risk factors for AIS. The combined prediction model utilizing clinical and imaging markers effectively predicts stroke risk in symptomatic ICAD patients.

## 1. Introduction

Intracranial atherosclerotic disease (ICAD) is a common cause of ischemic stroke in individuals of Asian descent (1), accounting for approximately 50% of strokes in China and Southeastern Asia (2). ICAD is a chronic and progressive disease that can affect multiple intracranial arteries simultaneously. Although patients may exhibit no clinical symptoms in the early stages of the disease, there is a risk of developing neurological deficits. As the disease progresses, transient ischemic attacks (TIAs) or even acute ischemic strokes (AISs) may occur. TIA and AIS can be considered successive manifestations of acute cerebrovascular events. Patients with TIA experience transient and sudden onset of focal neurological symptoms, which are usually quickly and completely relieved (3). TIA represents brain tissue hypoperfusion, and severe hypoperfusion can eventually result in irreversible ischemic necrosis of brain tissue, significantly increasing the disability and mortality risk for patients with ICAD (4). TIA is considered the most valuable early warning sign prior to the onset of stroke. Studies have shown that the proportion of TIA patients who experience a stroke within 90 days is as high as 20% (5, 6). In a large-scale international study involving 3,847 patients with TIA or minor strokes, it was found that the incidence of cardiovascular events (including stroke) was 6.4% in the first year and 6.4% from the second to fifth years (7). Yang et al. (8) discovered that asymptomatic intracranial arterial plaques are more stable than symptomatic intracranial arterial plaques, as quantified by the degree of plaque enhancement. Therefore, asymptomatic intracranial arterial plaques are less likely to rupture and block distal blood vessels, often resulting in less severe clinical consequences. Symptomatic ICAD indicates that patients have already experienced neurological deficits. Studies have shown that the annual stroke recurrence rate for symptomatic ICAD is as high as 12.6% (9), with a high likelihood of progressing to irreversible AIS. Thus, active prevention and treatment are necessary for these patients.

The Framingham Stroke Risk Profile (FSRP) is a commonly used scale for predicting the risk of stroke in the next decade (10). FSRP takes into account various clinical risk factors that can exacerbate arteriosclerosis and accelerate the progression of atherosclerosis. Additionally, high-resolution vascular wall imaging (HR-VWI) technology has been widely employed in the clinical diagnosis of ICAD, enabling the analysis of plaque composition, measurement of stenosis, assessment of remodeling patterns, and evaluation of plaque stability (11). Dynamic susceptibility contrast-enhanced perfusion imaging (DSC-PWI) can be utilized for qualitative analysis and quantitative measurement of ischemic lesions. Studies have demonstrated that PWI can identify more than 50% of ischemic lesions in patients with suspected hemispheric TIA (12). It can provide objective evidence for the diagnosis and treatment of symptomatic ICAD patients.

Risk assessment of stroke can significantly impact the management of risk factors. The pathogenesis of AIS involves perforating artery occlusion, arterial–arterial embolism, hypoperfusion, and mixed mechanisms (13), which encompass plaques and hypoperfusion. Most existing models used for predicting AIS only focus on clinical risk factors and macroangiopathy, neglecting the influence of cerebral perfusion status (14). Studies on TIA risk stratification often overlook the causes of macroangiopathy and solely concentrate on perfusion changes (15). Currently, clinical risk factor models combined with multimodal MRI (mMRI) do not consider the comprehensive impact of multiple clinical risk factors on ICAD (16); the FSRP score can address this limitation. The goal of this study is to develop a clinical-imaging model for predicting AIS utilizing mMRI in conjunction with the FSRP in patients with ICAD to achieve accurate risk stratification and early secondary stroke prevention.

## 2. Materials and methods

### 2.1. Patient population selection

This study is a prospective case-series study that continuously recruited patients with cerebral ischemic symptoms from the neurology department (including both outpatients and inpatients) at the First Affiliated Hospital of Harbin Medical University from January 2021 to June 2023. The study received approval from the Ethics Committee of the First Affiliated Hospital of Harbin Medical University (approval number 201989), and all patients or their guardians provided informed consent by signing the consent forms.

The inclusion criteria were as follows: (1) clinical diagnosis of TIA or AIS with symptoms of cerebral ischemia; (2) mMRI scan conducted within 7 days after the onset of symptoms; (3) presence of ICAD in the blood supply vessels of the ischemic areas; and (4) age ≥ 18 years old. The exclusion criteria were as follows: (1) cerebral ischemic changes caused by non-ICAD, such as moyamoya disease, arteritis, thromboembolism, etc.; (2) patients who have undergone brain surgery, experienced cerebral hemorrhage, or have cerebral tumors; (3) lesions that are inconsistent with the arterial blood supply area of the responsible artery; (4) contraindications for MRI; and (5) poor image quality that prevents proper analysis.

### 2.2. Scanning protocol

A 3.0 T MRI scanner (Ingenia Elition; Philips Healthcare, Best, the Netherlands).

with 32-channel head orthogonal coils was used. The scanning sequences and parameters were as follows: (1) Diffusion-weighted imaging (DWI): repetition time (TR) /echo time (TE) =.

2,851 ms/47 ms; thickness = 4 mm; Gap = 1 mm; field of view (FOV) = 230 mm × 230 mm × 119 mm; Voxel = 2.05 × 2.56; Matrix = 112 × 90; (2) Three-dimension time of flight magnetic resonance angiography (3D-TOF-MRA): TR/TE = 25 ms/3.5 ms; thickness = 1.4 mm; Gap = −0.7 mm; FOV = 194 mm × 194 mm × 84 mm; Voxel = 0.7 × 0.7; Matrix = 275 × 275; (3) T1-weighted three-dimension volumetric isotropic turbo spin echo acquisition (T1W-3D-VISTA): TR/TE = 800 ms/19 ms; thickness = 0.6 mm; Gap = −0.3 mm; FOV = 200 mm × 181 mm × 36 mm; Voxel = 0.6 × 0.6; Matrix = 332 × 300; (4) T2-weighted three-dimension volumetric isotropic turbo spin echo acquisition (T2W-3D-VISTA): TR/TE = 2,400 ms/90 ms; thickness = 0.6 mm; Gap = −0.3 mm; FOV = 200 mm × 200 mm × 40 mm; Voxel = 0.6 × 0.6; Matrix = 332 × 332; and (5) simultaneous non-contrast angiography and intraplaque hemorrhage (SNAP): TR/TE = 10 ms/5.8 ms; thickness = 0.6 mm; Gap = −0.3; FOV = 200 mm × 181 mm × 36 mm; Voxel = 0.8 × 0.8; Matrix = 200 × 196; (6) DSC-PWI: TR/TE = 1795/40 ms, thickness = 0.6 mm; Gap = 0 mm;FOV = 224 mm × 224 mm × 120 mm; Voxel = 2.33 × 2.33; Matrix = 96 × 94, T2*-EPI technology was used for image acquisition, and a contrast agent (gadolinium gluconate) was injected through the elbow vein with a high-pressure syringe at a dose of 0.1 mL/Kg and a speed of 5 mL/s, scanning 50 phases in total, and finally scanning after enhanced T1W-VISTA. The total scanning time was approximately 40 min.

### 2.3. Clinical data analysis

The clinical baseline characteristics and laboratory examinations of the patients were collected, including sex, age, height, weight, body mass index (BMI), presence of hypertension, diabetes, smoking history, drinking history, and previous stroke history. Additionally, the levels of systolic blood pressure (SBP), diastolic blood pressure (DBP), homocysteine (Hcy), total cholesterol, triglycerides, low-density lipoprotein (LDL), high-density lipoprotein (HDL), apoprotein (apo) A, and apoB were recorded.

FSRP score (17): The evaluation items include age, sex, SBP, smoking history, diabetes history, cardiovascular history, atrial fibrillation history, and left ventricular hypertrophy history. Based on age and sex, scores were assigned, and the total FSRP score was calculated by summing all the scores. The total score corresponds to the corresponding 10-year stroke risk (Supplementary Table S1). A risk of less than 10% is classified as low-medium risk, while a risk of 10% or higher is defined as high risk.

### 2.4. Image analysis

The image data were transferred to a Philips IntelliSpace Portal (ISP, V10; Philips Healthcare, Best, The Netherlands) workstation for analysis. All quantitative data were measured by two neuroradiologists, and the final results were the average of their assessments. In cases where there was any ambiguity in the qualitative data, a consensus was reached through discussion.

#### 2.4.1. HR-VWI

All patients were categorized into two groups, the AIS group and the TIA group, based on the presence or absence of diffusion-limited areas in the DWI images. An atherosclerotic plaque was defined as an eccentric thickening of the blood vessel wall. The responsible blood vessels were determined based on the patient’s clinical symptoms and the location of the DWI diffusion limitation. The location of the responsible plaque was defined as the plaque at the same layer as the lesion or at the adjacent upper/lower layer (18), or the plaque at the narrowest point of the blood vessel. The presence or absence of intraplaque hemorrhage (IPH) and plaque enhancement were analyzed, and the signal intensity (SI) of the plaque and brain gray matter before and after enhancement was measured. IPH was defined as an SI of the plaque that was greater than 1.5 times that of adjacent muscles on the T1W-3D-VISTA image before enhancement, and the SNAP image displayed a significantly high signal (19). Plaque enhancement was classified into three grades (grade 0: signal lower than or equal to the vascular wall before enhancement; grade 1: signal higher than the vascular wall before enhancement but lower than that of the pituitary stalk; and grade 2: signal higher than the pituitary stalk) (20). The standardized plaque index (SQI) and plaque enhancement ratio (ER) were calculated according to the following formulas: SQI = SI _post-plaque_/SI _post-gray matter_, ER = [SI _post-plaque_/SI _post-gray matter_ – SI _pre-plaque_/SI _pre-gray matter_]/(SI _pre-plaque_/SI _pre-gray matter_) (21). The lumen area (LA) and vascular area (VA) of the stenosis level and the reference level were measured, and the reference level selected the non-diseased area closest to the proximal end of the stenosis level. The wall area (WA = VA _MLN_- LA _MLN_) (16), normative wall index (NWI = WA _MLN_/VA _MLN_) (16), stenosis rate (Stenosis rate = [1- LA _MLN_/LA _reference_] × 100%) (22), and remodeling index (RI=VA _MLN_/VA _reference_) (23) were calculated. Remodeling index (RI) ≥ 1.5 was defined as positive remodeling (PR), 0.95 < RI < 1.05 was non-remodeling, and RI ≤ 0.95 was negative remodeling (NR) (23).

#### 2.4.2. DSC-PWI

According to the PWI source image, a pseudocolor image was generated for visual analysis of abnormal perfusion areas. Taking the diseased area as the region of interest (ROI), the contralateral region of interest was mirrored. The relative cerebral blood flow (rCBF), relative cerebral blood volume (rCBV), time to peak (TTP), and mean transit time (MTT) values were measured for the lesion area. The ROI size was approximately 15 mm^2^. If the patient did not exhibit a local abnormal perfusion area, the average perfusion parameters of the blood supply area of the responsible artery were measured. The average perfusion parameters of the anterior circulation included the average values of the frontal lobe (near the anterior horn of the lateral ventricle), parietal lobe (near the posterior horn of the lateral ventricle), temporal lobe (near the temporal horn of the lateral ventricle), hippocampus, and basal ganglia. The average perfusion parameters of the posterior circulation included the average values of the brainstem (midbrain, pons, and medulla oblongata levels) and the bilateral cerebellar hemispheres (fourth ventricle level).

### 2.5. Statistical analysis

Statistical analysis was conducted using IBM SPSS version 26.0. Count data were presented as numbers and percentages (n/%), and the chi-square (χ^2^) test was employed to compare count data between groups. The one-sample Kolmogorov–Smirnov test was used to assess the normality of continuous variables. If the data followed a normal distribution and exhibited homogeneous variances, they were expressed as mean ± standard deviation, and dependent sample t-tests were used for between-group comparisons. If the data did not conform to a normal distribution, they were represented as the median and interquartile range (IQR [Q1-Q3]), and the Mann–Whitney U test was used for between-group comparisons. Risk factors with a value of *p* <0.1 were included in multivariate logistic regression analysis to identify independent risk factors for AIS. Receiver operating characteristic (ROC) curves were constructed to determine the area under the curve (AUC). The diagnostic performance of different models’ ROC curves was compared using Delong’s test (MedCalc Statistical Software Version 22.007). R software (version 4.0.1) was utilized to generate the nomogram model and assess the clinical net benefit of the model. A value of *p* <0.05 was considered statistically significant.

## 3. Results

**Figure 1:**
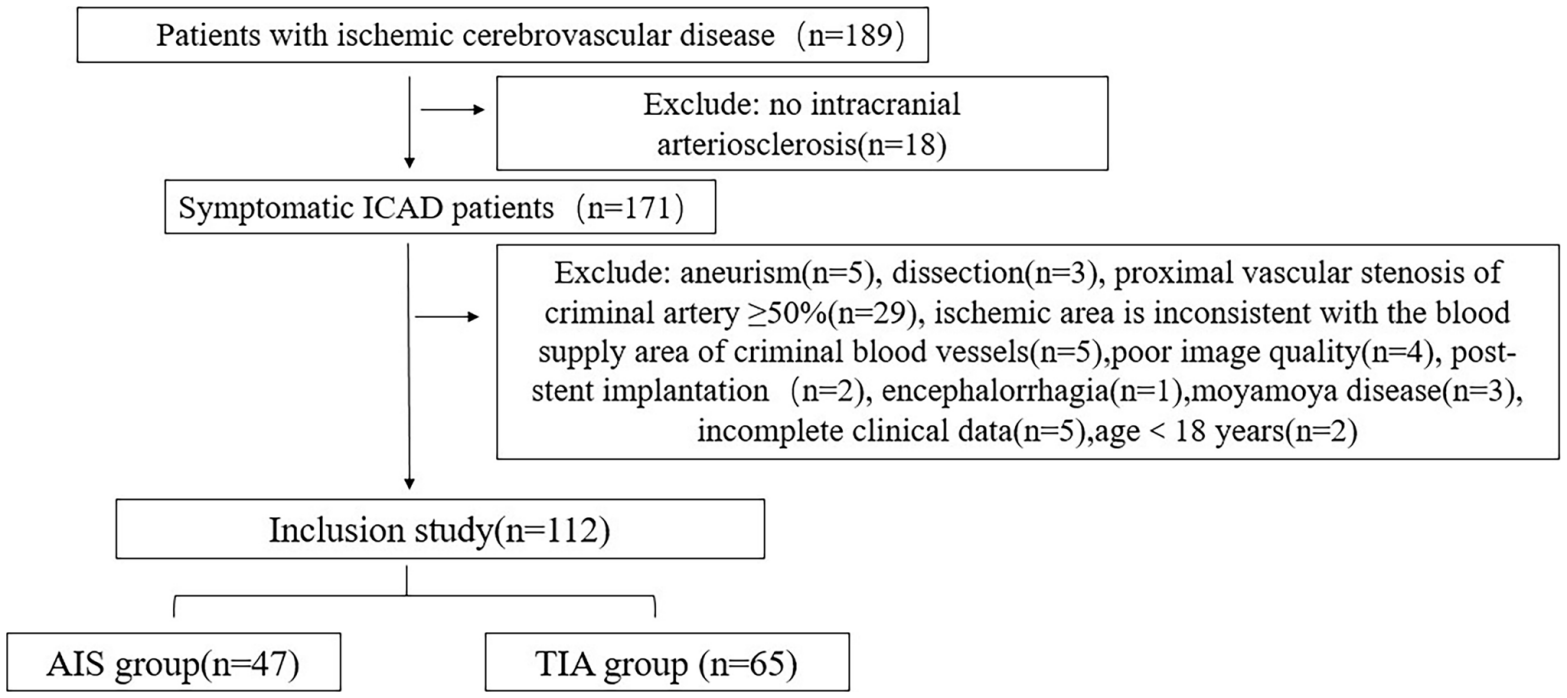
Flowchart of patient intake and discharge.

From January 2021 to May 2023, a total of 189 patients with symptomatic ICAD were enrolled in this study, while 77 patients who did not meet the research criteria were excluded. As a result, 112 patients were included in the final analysis. Among them, there were 65 patients (53.8% men and 46.2% women) in the TIA group, with an average age of 57.00 ± 11.50 years, and 47 patients (70.2% men and 29.8% women) in the AIS group, with an average age of 56.19 ± 11.31 years. Figure 1 shows the flowchart illustrating the patient recruitment and exclusion process.

### 3.1. Clinical data between the TIA group and AIS group

In the AIS group, the proportion of patients with hypercholesterolemia, high LDL levels, and elevated SBP was higher compared to the TIA group, and these differences were statistically significant (*p* < 0.05 for all) (Table 1). Additionally, the proportion of patients classified as FSRP-high risk was higher in the AIS group than in the TIA group (*p* = 0.01).

**Table 1:** Comparison of clinical data between the TIA group and AIS group.

	TIA group (*n* = 65)	AIS group (*n* = 47)	*x*^2^/t/Z	*p*
Male (*n*/%)	35/53.8	33/70.2	3.063^a^	0.08
Age (years)	57.00 ± 11.50	56.19 ± 11.31	-0.037^b^	0.712
Hypertension (*n*/%)	49/ 75.4%	38/ 80.9%	0.470^a^	0.493
SBP (mmHg)	133.00 (121.50, 143.50)	139.00 (125.00, 155.00)	−2.021^c^	0.043*
DBP (mmHg)	81.00 (77.50, 93.50)	85.00 (78.00, 95.00)	−1.205^c^	0.228
Diabetes (*n*/%)	23/ 35.4%	21/ 44.7%	0.988^a^	0.320
BMI (kg/m2)	25.27 ± 3.17	24.88 ± 3.09	0.655^b^	0.514
Hyperhomocysteinemia (*n*/%)	37/ 56.9	38/ 80.9	7.069^a^	0.008*
Hcy (umol/L)	10.44 (7.99, 12.50)	12.20 (10.30, 13.79)	−2.532^c^	0.011*
Cholesterol (mmol/L)	3.63 (3.17, 4.44)	4.13 (3.16, 4.66)	−1.350^c^	0.177
Triglyceride (mmol/L)	1.49 (1.10, 1.86)	1.46 (1.04, 2.08)	−0.301^c^	0.764
LDL (mmol/L)	2.03 (1.67, 2.56)	2.53 (1.99, 2.79)	−2.232^c^	0.026*
HDL (mmol/L)	1.03 ± 0.24	1.06 ± 0.20	−0.804^b^	0.423
L/H	2.29 ± 0. 69	2.38 ± 0.71	−0.733^b^	0.465
apoA (g/L)	1.19 (105, 1.30)	1.15 (1.00, 1.31)	−0.398^c^	0.691
apoB (g/L)	0.77 (0.59, 0.91)	0.83 (0.65, 1.01)	−1.454^c^	0.146
A/B	1.62 (1.33, 1.94)	1.37 (1.10, 1.87)	−1.722^c^	0.085
Smoking history (*n*/%)	23/ 35.4	22/4 6.8	1.481^a^	0.224
Drinking history (*n*/%)	10/ 15.4	9/ 19.1	0.274^a^	0.600
Stroke history (*n*/%)	15/ 23.1	13/ 27.1	0.306^a^	0.580
FSRP(high risk) (*n*/%)	24/ 36.9	29/ 61.7	6.718^a^	0.010*

^a^, *x*^2^ value; ^b^, *t* value; ^c^, *Z* value; *, the difference was statistically significant.

BMI, body mass index; Hcy: Homocysteine; LDL, low-density lipoprotein; HDL, high-density lipoprotein; FSRP, revised Framingham Stroke Risk Profile.

### 3.2. Comparison of vascular wall characteristics and perfusion parameters between the two groups

**Figure 2:**
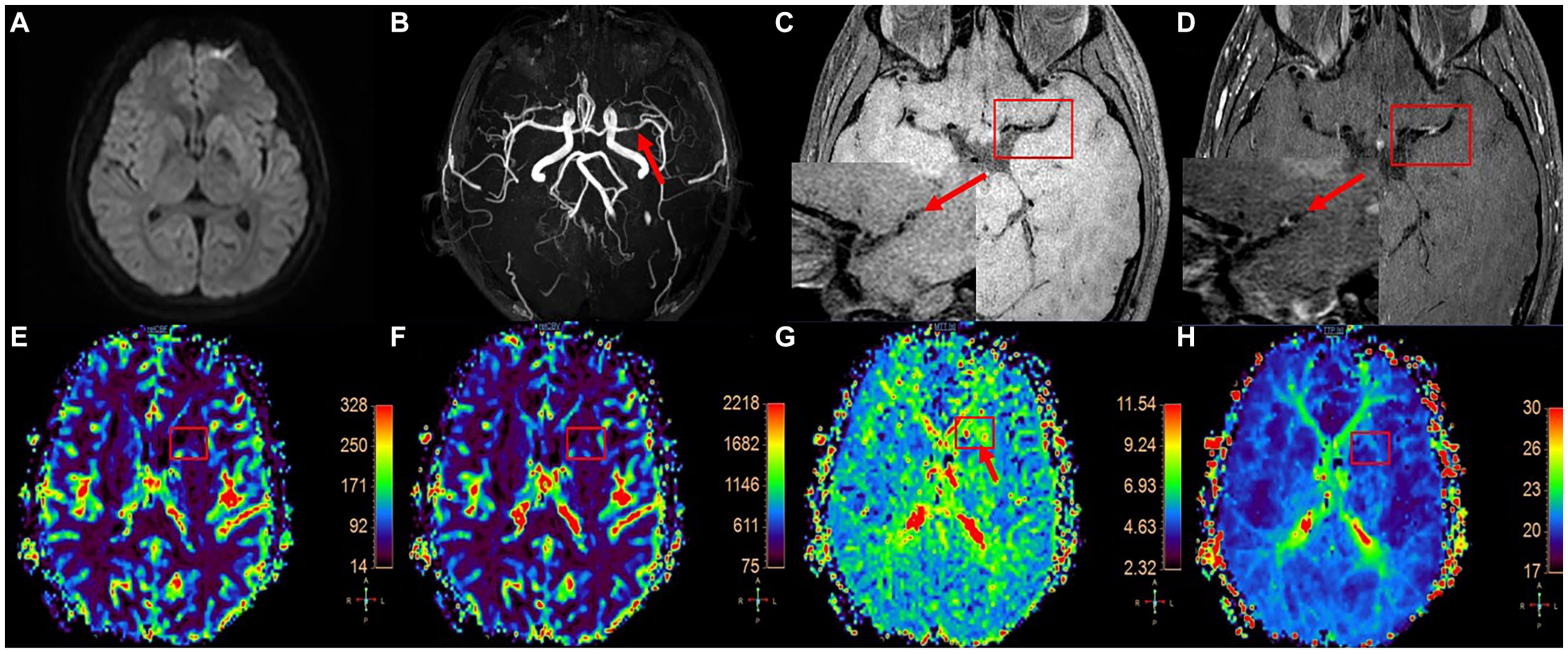
A patient with left MCA plaque was admitted to the hospital because of weakness in the right lower limb and was clinically diagnosed with anterior circulation TIA. DWI image shows that there is no diffusion restriction in the blood supply area of left MCA **(A)**, 3D-TOF-MRA shows local stenosis of left MCA (**B**, as shown by the arrow), T1W-VISTA image **(C)** shows thickening of MCA wall (short axis view of MCA in the lower left corner, as shown by the arrow) and plaque formation, and obvious enhancement can be seen in enhanced scanning **(D)**. DSC-PWI pseudocolor image **(E–H)** shows abnormal perfusion in the right basal ganglia.

It was observed that plaque enhancement was more pronounced in the AIS group, with higher values of SQI (*p* = 0.025) and ER (*p* = 0.029) compared to the TIA group. Furthermore, the MTT (*p* = 0.005) and TTP (*p* = 0.001) of the lesion area were longer in the AIS group than in the TIA group. There were no significant differences in other plaque characteristics and cerebral perfusion parameters between the two groups (Table 2; Figure 2).

**Table 2:** Comparison of vascular wall characteristics and perfusion parameters between the TIA group and AIS group.

	TIA group (*n* = 65)	AIS group (*n* = 47)	*x*^2^/t/Z	*p*
IPH (*n*/%)	22/ 33.8	15/ 31.9	0.046^a^	0.830
SQI	1.36 ± 0.41	1.55 ± 0.46	−2.268^b^	0.025*
ER	0.63 ± 0.51	0.85 ± 0.54	−2.215^b^	0.029*
Enhancement grade (*n*/%)			5.874^a^	0.053
0	12/ 18.5	2/ 4.3		
1	45/ 69.2	35/ 74.5		
2	8/ 12.3	10/ 21.3		
WA	4.67 (3.46, 6.87)	5.23 (3.00, 6.92)	−0.024^c^	0.981
NWI	0.84 (0.74, 0.91)	0.83 (0.54, 0.93)	−0.027^c^	0.979
Remodeling mode (*n*/%)			0.700^a^	0.403
PR Non-PR	28/ 43.1 37/ 56.9	24/ 51.1 23/ 48.9		
Stenosis rate	0.73 (0.45, 0.88)	0.74 (0.46, 0.92)	0.027^c^	0.979
Perfusion of lesion area				
rCBF	39.59 (25.02, 51.16)	30.62 (12.06, 43.68)	−1.630^c^	0.103
rCBV	436.83 (296.66, 552.53)	403.96 (194.73, 653.36)	−0.345^c^	0.730
MTT (s)	11.10 (8.99, 15. 52)	15.20 (10.18, 19.96)	−2.792^c^	0.005*
TPP (s)	25.19 (21.92, 28.85)	28.49 (25.93, 34.79)	−3.381^c^	0.001*

^a^, *x*^2^ value; ^b^, *t* value; ^c^, *Z* value; *, the difference was statistically significant.

IPH, intraplaque hemorrhage; SQI, standardized plaque index; ER, enhancement ratio; WA, wall area; NWI, normative wall index; RI, remodeling index; rCBF, relative cerebral blood flow; rCBV, relative cerebral blood volume; MTT, mean transit time; TPP, time to peak.

### 3.3. Multivariate logistics analysis to determine the independent risk factors leading to AIS

**Figure 3:**
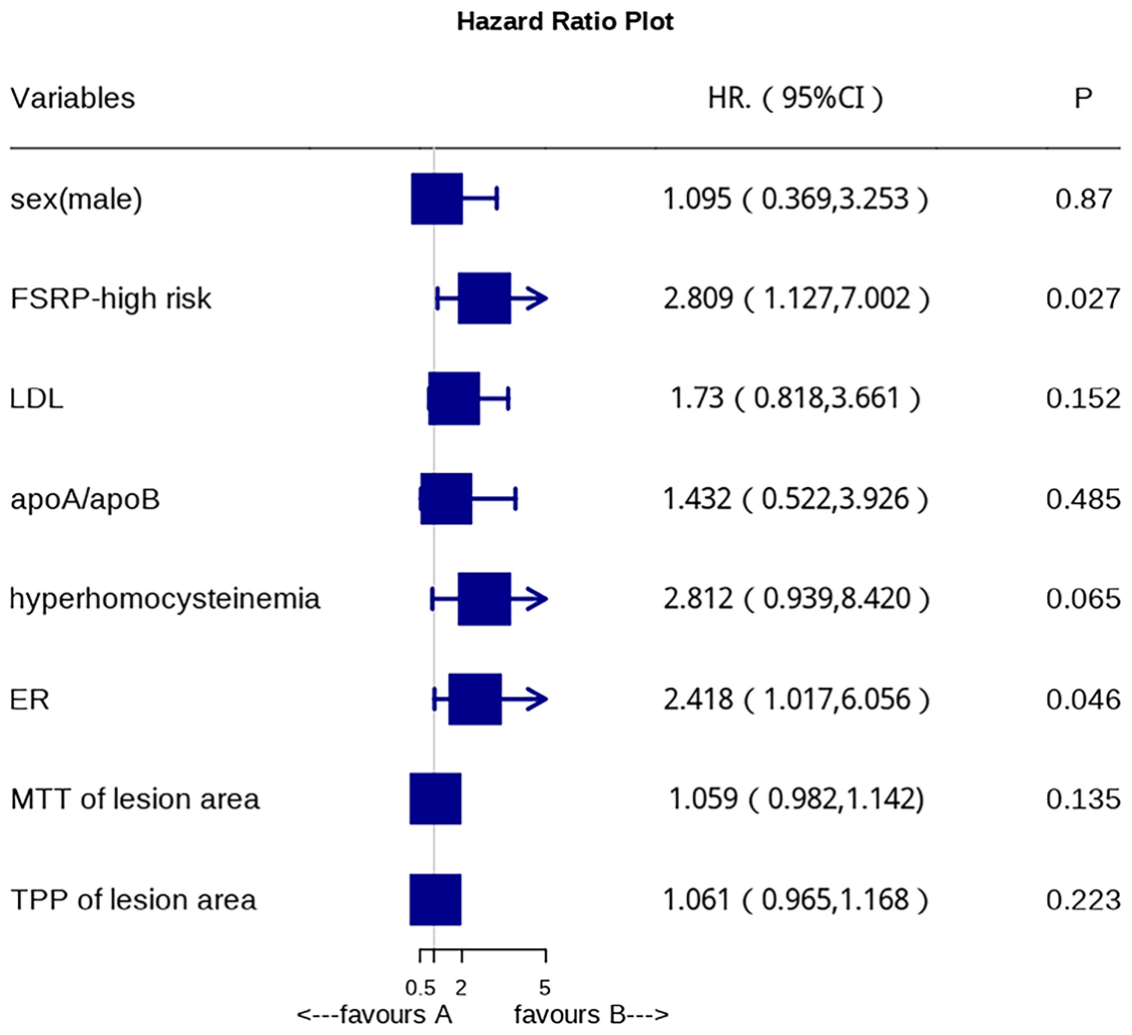
Forest map shows the independent risk factors of AIS.

After adjusting for confounding factors, multivariate logistic regression analysis revealed that FSRP-high risk and high ER (*p* = 0.046) are independent risk factors for AIS (Figure 3).

### 3.4. Establishment of AIS prediction models

**Figure 4:**
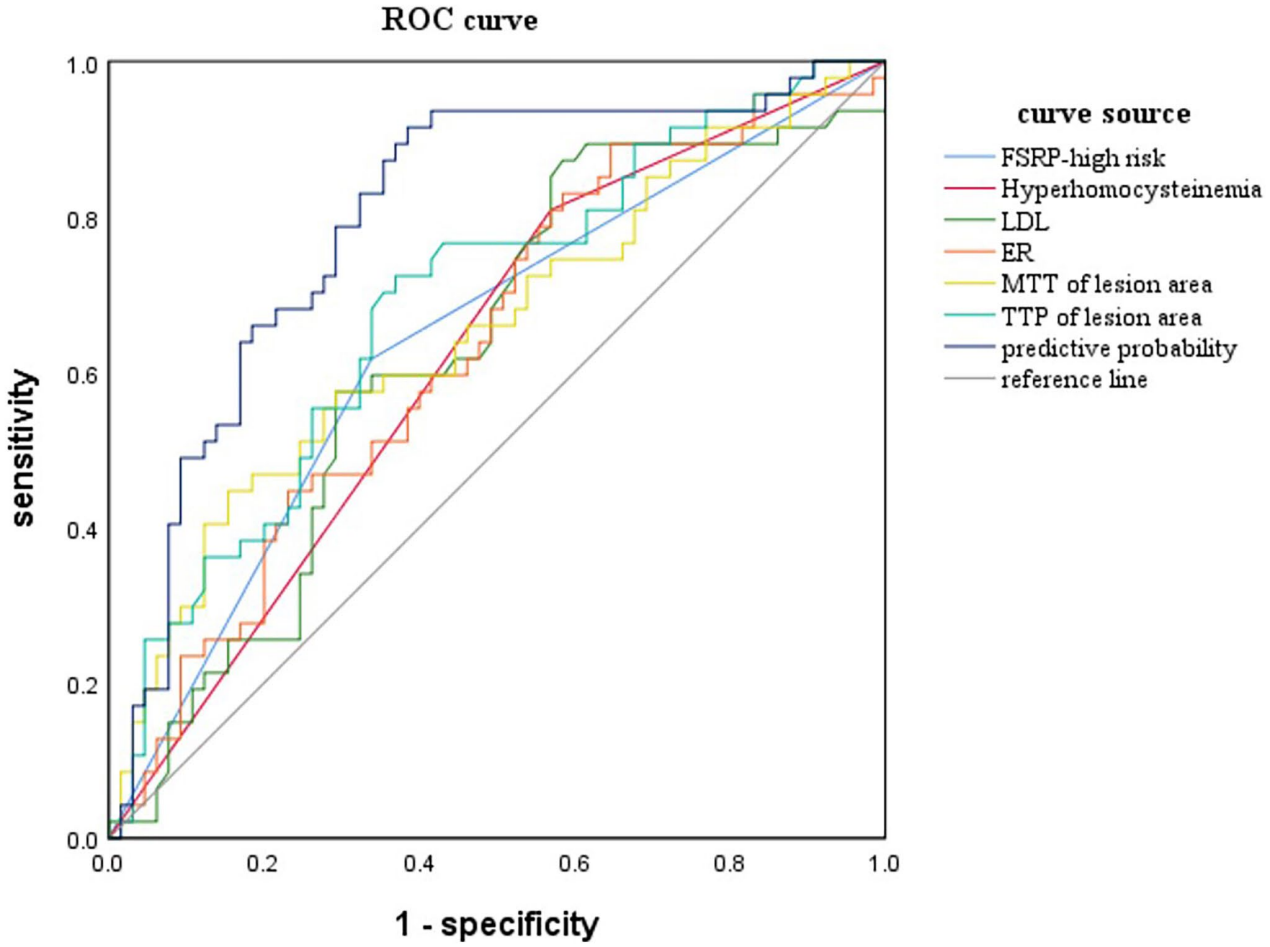
ROC curve for predicting AIS is constructed by combining clinical risk factors and imaging features.

The variables with *p* < 0.05 in the univariate analysis were included in the logistic regression analysis to construct the ROC curve (Figure 4). When combining the ROC curve with various clinical risk factors and imaging markers, the area under the curve (AUC) was 0.791, with a sensitivity of 85.11% and specificity of 66.15%. The results of the Delong test indicated that the diagnostic efficiency of this combined ROC curve was superior to that of ROC curves constructed using each variable alone, and the differences were statistically significant (*p* < 0.05).

**Figure 5:**
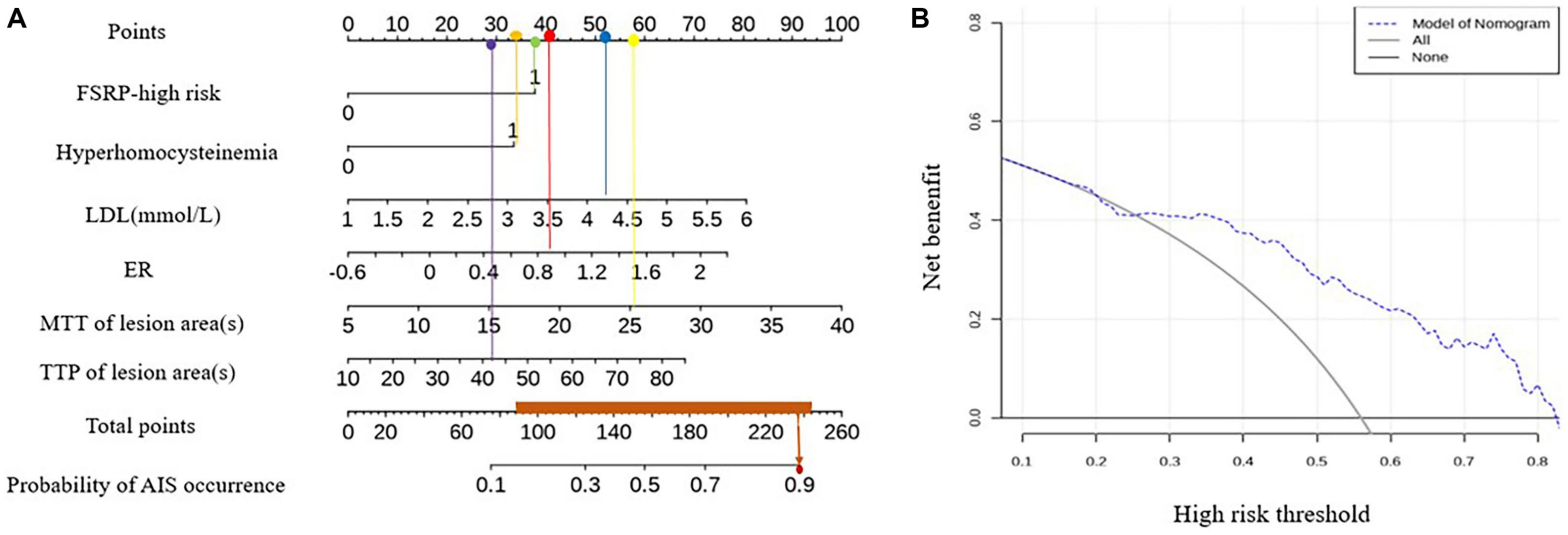
Nomogram model and DCA curve for predicting AIS.

An example of using the nomogram model to determine the probability of AIS in a specific patient is illustrated in Figure 5. The total score is calculated by summing the scores of individual variables using the nomogram, and the corresponding incidence probability indicates the likelihood of AIS in a specific patient with ICAD. The decision curve analysis (DCA) curve demonstrates that the model can generate better clinical net benefits when the threshold probability ranges from 0.16 to 0.82 (Figure 5).

## 4. Discussion

The study applied mMRI and traditional clinical risk factors to analyze the clinical and imaging markers in patients with ICAD. The results showed that in the AIS group, the proportion of FSRP-high risk, hyperhomocysteinemia, LDL, SQI, and ER were higher compared to the TIA group. Additionally, the MTT and TTP values in the lesion area of AIS patients were significantly longer than those in the TIA group. FSRP-high risk and higher ER were identified as independent risk factors for AIS. ROC and nomogram models combining clinical and imaging features were established, demonstrating good diagnostic efficacy and clinical practicability in predicting the possibility of AIS in patients with ICAD.

This study revealed that FSRP-high risk is an independent risk factor for AIS, indicating a higher burden of cardiovascular risk factors. The evaluation criteria of FSRP encompass various cardiovascular risk factors, which may increase the incidence of dominant cerebrovascular diseases, including stroke, through their detrimental effects on neurovascular function (24). Notably, the SBP in AIS patients was significantly higher than that in TIA patients, and increased SBP has been linked to an elevated risk of cardiovascular events-related mortality (25, 26). High Hcy levels, hypertension, and hyperhomocysteinemia are recognized as traditional cardiovascular risk factors and the study found that in AIS patients, the combination of high Hcy levels and hypertension can double the risk of cardiovascular disease (27). Moreover, the study revealed higher LDL levels in the AIS group compared to the TIA group. LDL, as a key factor in plaque lipid core, plays a crucial role in atherosclerotic cardiovascular disease (ASCVD) induction and is a primary target for ASCVD prevention (28). Therefore, relying solely on FSRP for stroke risk prediction may not be sufficient. Evaluating Hcy and blood lipid levels as complementary factors to FSRP can provide clinicians with a more comprehensive assessment of stroke risk in patients, enabling targeted secondary prevention measures.

The secondary prevention of ischemic stroke relies on identifying the cause of the initial stroke (29). The clinical application of HR-VWI and PWI has improved the ability to diagnose the etiology of patients with ICAD, including macroangiopathy and microcirculation ischemia. The study showed that the SQI and ER of plaque were higher in the AIS group compared to the TIA group. SQI and ER are quantitative indices representing plaque enhancement, which aligns with the findings of this study. Numerous studies have confirmed that enhanced intracranial atherosclerosis plaques are associated with recent ischemic events and can serve as indicators of plaque stability (8, 30, 31). Therefore, anti-inflammatory interventions may be the optimal clinical approach for stabilizing plaques and preventing AIS in ICAD patients with significantly enhanced plaques. The study also found that the extension of MTT and TTP was more significant in the AIS group compared to the TIA group, while no differences were observed in rCBF and rCBV between the two groups. MTT and TTP are time parameters, and their increased values indicate a slowdown in blood flow and decreased perfusion levels in brain tissue. This change occurs before the decrease in rCBF and rCBV, reflecting adjustments in cerebral hemodynamic reserve (32). Therefore, MTT and TTP serve as more sensitive indicators for detecting abnormal cerebral perfusion, assisting clinicians in identifying subclinical cerebral ischemia and providing timely treatment to improve microcirculation, ultimately preventing persistent cerebral hypoperfusion and stroke.

Previous studies have focused on comparing risk factors between AIS and non-AIS individuals. Non-AIS individuals often include asymptomatic volunteers, making it challenging to identify them in clinical practice unless they undergo physical examinations. This study emphasizes patients with symptomatic cerebral ischemia, who are considered to be at high risk of stroke, making it crucial to evaluate their AIS risk and implement effective prevention strategies.

Based on the clinical and imaging risk factors identified for AIS, a ROC model was established to predict AIS with an AUC value of 0.791, sensitivity of 85.11%, and specificity of 66.15%. Currently, numerous studies have been conducted to predict future stroke risk in TIA patients using various scoring and imaging techniques. Hayashi T et al. found that the AUC of ABCD2 in predicting future stroke in TIA patients was 0.662 (33). They developed a prediction model based on age, hemiplegia, macrovascular risk characteristics, and previous cerebral infarction, which improved the prediction ability of TIA stroke risk with an AUC value of 0.804. In contrast to our study, Hay T et al. only studied arterial lesions >50% as variable values, while our study did not limit the stenosis degree of the responsible artery, as even mild stenosis may be accompanied by unstable plaques. Ren et al. (34) established a prediction model for the recurrence of symptomatic ICAD patients (including AIS and TIA) by combining clinical risk factors and plaque enhancement, achieving an AUC value of only 0.685. Although their predicted recurrence population differed from our study, their results indicated limited predictive capability for ICAD recurrence using clinical risk factors and plaque enhancement alone. Previous studies have demonstrated a relationship between perfusion defects and ipsilateral AIS in TIA patients (35), suggesting that the addition of perfusion parameters to the model could further improve prediction performance. Furthermore, to facilitate a more intuitive and convenient assessment of stroke risk in specific patients with ICAD, we developed a nomogram chart that demonstrates superior clinical net benefits by enhancing the efficiency of clinical decision-making, avoiding inadequate or excessive medical treatment, and facilitating early clinical intervention for patients with ICAD, and, therefore, reducing the risk of AIS.

There are several limitations to this study. First, the sample size was small, so there is a need for larger prospective studies to further validate the findings. Second, Tmax, one of the commonly used perfusion parameters with high sensitivity in evaluating changes in cerebral perfusion, was not obtained in this study. Calculating Tmax requires expensive commercial software, making it inaccessible for many smaller medical institutions. Therefore, we believe that rCBF, rCBV, MTT, and TTP are more practical parameters than Tmax. Lastly, long-term patient follow-up is essential to verify the model’s ability to distinguish stroke. As we did not conduct long-term follow-ups of symptomatic ICAD patients, future investigations will focus on the prognosis and recurrence in these patients.

## 5. Conclusion

FSRP-high risk and high ER are identified as independent risk factors for AIS. The combined prediction model, integrating clinical and imaging markers, can effectively predict stroke risk in symptomatic ICAD patients.

## Data availability statement

The raw data supporting the conclusions of this article will be made available by the authors, without undue reservation.

## Ethics statement

The studies involving humans were approved by Ethics Committee of the First Affiliated Hospital of Harbin Medical University. The studies were conducted in accordance with the local legislation and institutional requirements. The participants provided their written informed consent to participate in this study.

## Author contributions

JS: Formal analysis, Investigation, Methodology, Software, Writing – original draft. YS: Methodology, Formal analysis, Writing – original draft. YC: Investigation, Software, Writing – original draft. JL was responsible for technical guidance and manuscript revision. WW: Data curation, Project administration, Writing – review & editing, Validation.

## References

[1] Wang J Zhang S Lu J Qi P Hu S Yang X. High-resolution MR for follow-up of intracranial steno-occlusive disease treated by endovascular treatment. Front Neurol. (2021) 12:706645. doi: 10.3389/fneur.2021.706645 35002907 PMC8740140

[2] Romano JG Prabhakaran S Nizam A Feldmann E Sangha R Cotsonis G. Infarct recurrence in intracranial atherosclerosis: results from the MyRIAD study. J Stroke Cerebrovasc Dis. (2021) 30:105504. doi: 10.1016/j.jstrokecerebrovasdis.2020.105504 33276302 PMC7856286

[3] Clissold B Phan TG Ly J Singhal S Srikanth V Ma H. Current aspects of TIA management. J Clin Neurosci. (2020) 72:20–5. doi: 10.1016/j.jocn.2019.12.032 31911111

[4] Jiang L Zhou L Zhang H Geng W Yong W Cui J. MRI predictors of intracranial hemorrhage in acute ischemic stroke after endovascular thrombectomy therapy. Am J Transl Res. (2020) 12:4532–41. 32913526 PMC7476153

[5] Fitzpatrick T Gocan S Wang CQ Hamel C Bourgoin A Dowlatshahi D. How do neurologists diagnose transient ischemic attack: a systematic review. Int J Stroke. (2019) 14:115–24. doi: 10.1177/1747493018816430 30507363 PMC6604401

[6] Amarenco P Lavallée PC Monteiro Tavares L Labreuche J Albers GW Abboud H. Five-year risk of stroke after TIA or minor ischemic stroke. N Engl J Med. (2018) 378:2182–90. doi: 10.1056/NEJMoa1802712 29766771

[7] Wussler D Du Fay de Lavallaz J Mueller C. Five-year risk of stroke after TIA or minor ischemic stroke. N Engl J Med. (2018) 379:1580. doi: 10.1056/NEJMc1808913 30338963

[8] Yang WJ Abrigo J Soo YOY Wong S Wong KS Leung TWH. Regression of plaque enhancement within symptomatic middle cerebral artery atherosclerosis: a high-resolution MRI study. Front Neurol. (2020) 11:755. doi: 10.3389/fneur.2020.00755 32849214 PMC7399098

[9] Zhai SJ Jia L Kukun HJ Wang YL Wang H Ding S. Predictive power of high-resolution vessel wall magnetic resonance imaging in ischemic stroke. Am J Transl Res. (2022) 14:664–71. PMID: 35173884 PMC8829603

[10] Os D Ikram MA MJG L Ikram MK. The revised Framingham stroke risk profile in a primary prevention population: the Rotterdam study. Circulation. (2017) 135:2207–9. doi: 10.1161/CIRCULATIONAHA.117.028429 28559499

[11] Qiao Y Steinman DA Qin Q Etesami M Schär M Astor BC. Intracranial arterial wall imaging using three-dimensional high isotropic resolution black blood MRI at 3.0 tesla. J Magn Reson Imaging. (2011) 34:22–30. doi: 10.1002/jmri.22592 21698704

[12] Olivot JM Mlynash M Thijs VN Kemp S Lansberg MG Wechsler L. Optimal Tmax threshold for predicting penumbral tissue in acute stroke. Stroke. (2009) 40:469–75. doi: 10.1161/STROKEAHA.108.526954 19109547 PMC2670783

[13] Chen PH Gao S Wang YJ Xu AD Li YS Wang D. Classifying ischemic stroke, from TOAST to CISS. CNS Neurosci Ther. (2012) 18:452–6. doi: 10.1111/j.1755-5949.2011.00292.x 22268862 PMC6493455

[14] Montes D Vranic J Lim JC Song JW Silverman SB González RG. Cardiovascular risk factors affect specific segments of the intracranial vasculature in high-resolution (HR) Vessel Wall imaging (VWI). J Stroke Cerebrovasc Dis. (2021) 30:106026. doi: 10.1016/j.jstrokecerebrovasdis.2021.106026 34407497

[15] Wang Y Xiao J Luo Y Wang S Liang H Jin L. Risk factors of perfusion and diffusion abnormalities on MRI in hemispheric TIA: a case-control study. Ann Transl Med. (2019) 7:808. doi: 10.21037/atm.2019.12.69 32042824 PMC6989882

[16] Liu S Luo Y Wang C Tang R Sheng Z Xie W. Combination of plaque characteristics, Pial collaterals, and hypertension contributes to misery perfusion in patients with symptomatic middle cerebral artery stenosis. J Magn Reson Imaging. (2020) 51:195–204. doi: 10.1002/jmri.26778 31069889

[17] Pelcher I Puzo C Tripodis Y Aparicio HJ Steinberg EG Phelps A. Revised Framingham stroke risk profile: association with cognitive status and MRI-derived volumetric measures. J Alzheimers Dis. (2020) 78:1393–408. doi: 10.3233/JAD-200803 33164933 PMC7887636

[18] Sun J Liu G Zhang D Wu Z Liu J Wang W. The longitudinal distribution and stability of curved basilar artery plaque: a study based on HR-MRI. J Atheroscler Thromb. (2021) 28:1333–9. doi: 10.5551/jat.62448 33642443 PMC8629706

[19] Xu XH Liu CL Guo ZY. Clinical observation on the application of intracranial thrombectomy stent in the treatment of elderly patients with acute ischemic stroke. Cardiovascular and Cerebrovascular Disease Prevention and Treatment. (2021) 21:595–7. doi: 10.3969/j.issn.1009-816x.2021.06.021

[20] Qiao Y Zeiler SR Mirbagheri S Leigh R Urrutia V Wityk R. Intracranial plaque enhancement in patients with cerebrovascular events on high-spatial-resolution MR images. Radiology. (2014) 271:534–42. doi: 10.1148/radiol.13122812 24475850 PMC4263625

[21] Lin GH Song JX Huang TD Fu NX Zhong LL. Relationship between the stroke mechanism of symptomatic middle cerebral artery atherosclerotic diseases and culprit plaques based on high-resolution vessel wall imaging. Front Neurol. (2022) 13:968417. doi: 10.3389/fneur.2022.968417 36188409 PMC9523534

[22] Millon A Boussel L Brevet M Mathevet JL Canet-Soulas E Mory C. Clinical and histological significance of gadolinium enhancement in carotid atherosclerotic plaque. Stroke. (2012) 43:3023–8. doi: 10.1161/STROKEAHA.112.662692 22923447

[23] Qiao Y Anwar Z Intrapiromkul J Liu L Zeiler SR Leigh R. Patterns and implications of intracranial arterial remodeling in stroke patients. Stroke. (2016) 47:434–40. doi: 10.1161/STROKEAHA.115.009955 26742795 PMC4729583

[24] Iadecola C Parikh NS. Framingham general cardiovascular risk score and cognitive impairment: the power of foresight. J Am Coll Cardiol. (2020) 75:2535–7. doi: 10.1016/j.jacc.2020.03.061 32439002 PMC7853244

[25] Williams B Mancia G Spiering W Rosei EA Azizi M Burnier M. 2018 ESC/ESH guidelines for the management of arterial hypertension [published correction appears in Eur heart J. 2019;40(5):475]. Eur Heart J. (2018) 39:3021–104. doi: 10.1093/eurheartj/ehy30165516

[26] Jung MH Yi SW An SJ Yi JJ. Age-specific associations between systolic blood pressure and cardiovascular mortality. Heart. (2019) 105:1070–7. doi: 10.1136/heartjnl-2019-314697 31055498

[27] Graham IM Daly LE Refsum HM Robinson K Brattström LE Ueland PM. Plasma homocysteine as a risk factor for vascular disease.The European concerted action project[J]. JAMA. (1997) 277:1775–81. doi: 10.1001/jama.1997.03540460039030 9178790

[28] National Cholesterol Education Program Expert Panel on detection evaluation, treatment of high blood cholesterol in adults. Third report of the national cholesterol education program (NCEP) expert panel on detection, evaluation, and treatment of high blood cholesterol in adults (adult treatment panel III) final report. Circulation. (2002) 106:3143–421. 12485966

[29] Schaafsma JD Rawal S Coutinho JM Rasheedi J Mikulis DJ Jaigobin C. Diagnostic impact of intracranial Vessel Wall MRI in 205 patients with ischemic stroke or TIA. AJNR Am J Neuroradiol. (2019) 40:1701–6. doi: 10.3174/ajnr.A6202 31488500 PMC7028571

[30] Wang E Shao S Li S Yan P Xiang Y Wang X. A high-resolution MRI study of the relationship between plaque enhancement and ischemic stroke events in patients with intracranial atherosclerotic stenosis. Front Neurol. (2019) 9:1154. doi: 10.3389/fneur.2018.01154 30671018 PMC6331481

[31] de Havenon A Mossa-Basha M Shah L Kim SE Park M Parker D. High-resolution vessel wall MRI for the evaluation of intracranial atherosclerotic disease. Neuroradiology. (2017) 59:1193–202. doi: 10.1007/s00234-017-1925-9 28942481

[32] Walter U Kolbaske S Patejdl R Steinhagen V Abu-Mugheisib M Grossmann A. Insular stroke is associated with acute sympathetic hyperactivation and immunodepression. Eur J Neurol. (2013) 20:153–9. doi: 10.1111/j.1468-1331.2012.03818.x 22834894

[33] Hayashi T Kato Y Nagoya H Ohe Y Deguchi I Fukuoka T. Prediction of ischemic stroke in patients with tissue-defined transient ischemic attack. J Stroke Cerebrovasc Dis. (2014) 23:1368–73. doi: 10.1016/j.jstrokecerebrovasdis.2013.11.020 24389377

[34] Ren K Jiang H Li T Qian C Gong S Wang T. Predictive value of the combination between the intracranial arterial responsible plaque characteristics and the Essen stroke risk score for short-term stroke recurrence. J Stroke Cerebrovasc Dis. (2022) 31:106624. doi: 10.1016/j.jstrokecerebrovasdis.2022.106624 35933933

[35] Nam KW Kim CK Ko SB Yoon BW Yoo RE Sohn CH. Regional arterial spin Labeling perfusion defect is associated with early ischemic recurrence in patients with a transient ischemic attack. Stroke. (2020) 51:186–92. doi: 10.1161/STROKE 31718505

