# Supplementary material for: Predicting acute ischemic stroke using the revised Framingham stroke risk profile and multimodal magnetic resonance imaging
Source: Front Neurol. 2023 Sep 28;14:1264791. doi: 10.3389/fneur.2023.1264791 (PMC10568328; doi:10.3389/fneur.2023.1264791)
Supplement: Supplementary file 1 [file Data_Sheet_1.PDF]

Male Revised Framingham Stroke Risk Profile (FSRP) score

Scores	age （years）	systolic blood pressure before treatment （mmHg）	systolic blood pressure after treatment （mmHg）	diabetes	smoke	cardiovascular disease	Atrial fibrillation	left ventricular hypertrophy
0	＜56	97-105	97-105	No	No	No	No	No
1	57-59	106-115	106-112					
2	60-62	116-125	113-117	Yes				
3	63-65	126-135	118-123		Yes			
4	66-68	136-145	124-129			Yes	Yes	
5	69-72	146-155	130-135					Yes
6	73-75	156-165	136-142					
7	76-78	166-175	143-150					
8	79-81	176-185	151-161					
9	82-84	186-195	162-176					
10	＞85	196-205	177-205					

The 10-year stroke risk in male

Scores	1	2	3	4	5	6	7	8	9	10	11	12	13	14	15	16	17	18	19
risk (%)	3	3	4	4	5	5	6	7	8	10	11	13	15	17	20	22	26	29	33
Scores	20	21	22	23	24	25	26	27	28	29	30								
risk (%)	37	42	47	52	57	63	68	74	79	84	88								

Female Revised Framingham Stroke Risk Profile (FSRP). score

Scores	age （years）	systolic blood pressure before treatment （mmHg）	systolic blood pressure after treatment （mmHg）	diabetes	smoke	cardiovascular disease	Atrial fibrillation	left ventricular hypertrophy
0	＜56			No	No	No	No	No
1	57-59	95-106	95-106					
2	60-62	107-118	107-113			Yes		
3	63-64	119-130	114-119	Yes	Yes			
4	65-67	131-143	120-125					Yes
5	68-70	144-155	126-131					
6	71-73	156-167	132-139				Yes	
7	74-76	168-180	140-148					
8	77-78	181-192	149-160					
9	79-81	193-204	161-204					
10	82-84	205-216	205-216					

The 10-year stroke risk in female

Scores	1	2	3	4	5	6	7	8	9	10	11	12	13	14	15	16	17	18	19	20	21	22	23	24	25	26	27
risk (%)	1	1	2	2	2	3	4	4	5	6	8	9	11	13	16	19	23	27	32	37	43	50	57	64	71	78	84
